# Pleasant Thoughts for Tobacco Users

**Published:** 1868-06

**Authors:** 


PLEASANT THOUGHTS FOR TOBACCO USERS.—Dr. W. H. Wetherbee, in an article on the Chemistry of Tobacco, in the *Journal of Applied Chemistry*, imparts the following agreeable information to those who delight in the Indian weed.

Among the various adulterations to which chewing tobacco is subject, are lead, copper, antimony, copperas, black hellebore, alum, sugar or molasses, dock leaves, and corrosive sublimate. These are added to give flavor or pungency to the weaker and poorer varieties, and the legitimate effects of such villainous compounds need not be mentioned. Too much of the "fine-cut chewing tobacco" is wrapped in an inferior kind of tin-foil, having a great proportion of lead in its composition, and partial paralysis of the tongue and muscles of the mouth has followed its use, from the lead salts thus formed, when the simple use of tobacco in its pure state would have failed to produce results so deleterious.

For chewing purposes, an article called British herb tobacco has been substituted for the genuine "weed," and is composed of thyme, marjoram, and hyssop, of each two ounces; coltsfoot three ounces; betony and eyebright, of each four ounces; rosemary and lavender, of each eight ounces; the whole mixed, pressed together, and cut in the form of plug tobacco. It is harmless, cheap, and among the poorer classes answers a good purpose. For smoking purposes, the bark of the cascarilla is sometimes added to impart a peculiar flavor, and the leaves of various other plants are sometimes substituted in part for those of the tobacco. Nitre is sometimes added to make it burn more rapidly, though it is frequently found in small quantities as a product of the chemical process of curing the plant Potash, as before observed, exists already formed as one of the component parts of tobacco, aside from the trace of the nitrate which it contains, and in the usual process of preparation a weak solution of potash, or its carbonate, is also sprinkled upon the leaves, and it is not unlikely, that while being dried, they may absorb a portion of nitrogen and oxygen from the atmosphere, sufficient to form a small amount of the nitrate.

Snuff-taking, though at one time almost a universal practice, has now fallen greatly into disuse, though in some of the Southern and Western States, and among the Spanish dames of the West Indies, the disgusting habit of *chewing snuff*, or, as it is called, *dipping*, is still followed to a great extent. Medicinally, it is recommended for colds, catarrh in the head, and several other purposes. Snuff is usually adulterated with salt, for the purpose of increasing its weight and keeping it moist, and with urine, muriate of ammonia, and powdered glass, to increase the acrimony and pungency. Some kinds are moistened with cane-juice, or molasses and water, which give rise to the vinous fermentation, and rum is sometimes added to produce the same flavor. Quick-lime or caustic alkali is sometimes added to the tobacco to develope the flavor, as well as to neutralize the acid formed in fermentation.

Many other substances are also added, either to color or flavor, thus forming the different varieties of Scotch, Welsh, Spanish, Lundyfoot, French, Russian, Strasburgh, Maccaboy, and many others. They are sometimes medicated with subsulphate of mercury; nitrate of silver, etc., for catarrh, headache, inflammation of the eyes, and other diseases of the nerves of the head, the mucous membrane of the nose, etc.

